# Health Tract, No. 238

**Published:** 1866-05

**Authors:** 


From the Boston Watchman and Reflector, by Dr. W. W. Hall, M.D.

HEALTH TRACT, No. 238.

### PREACHING EASILY.

No physiological fact is more clearly established than that a night of good sleep rests the body and invigorates the brain; hence, persons in ordinary health after sleeping soundly arise from their beds in the morning with an amount of bodily and mental power proportioned to the time and healthfulness of the sleep. Extensive medical observation shows also that, whether in animals or men, sleep is most nutritious, most invigorating, when taken during the two or three hours before and after midnight. No one denies that it is a clergyman's duty to use these indisputable facts practically. The first step then to be taken by a faithful and earnest worker in the ministry, as a means of enabling him to make the most of every Sabbath day, is to go to bed about nine o'clock on Saturday evening, for he has no right to intrench on the hours of God's day in preparation for the active work of that day. He should not go to sleep after waking up in the morning, if it is day-light; nor is it best to get up at once, but to remain in bed until there is a feeling of rest all over the body, and as if it would be a relief to get up and wash and dress. Having secured a good degree of vigor with which to begin the Sabbath day's work, he should use it economically, wisely; he should husband his strength, by not putting it forth unnecessarily nor lavishly on the earlier service, but seek to distribute it over the whole work of the day. If all the "vim" is exhausted on the mornings discourse, both preacher and people will necessarily be over-sleepy in the afternoon, and half a Sabbath, with its glorious and fleeting privileges, is lost forever.

Every word uttered, every note sounded, even the crook of a finger or wink of the eye, is at the cost of power; a wink is not much, but a dozen or two winks in quick succession produces appreciable fatigue or tiredness. Hence a clergyman will speak easier, if, until he enters the pulpit, he does not speak a sentence, or sing a line, or make a nod. And even if he takes his breakfast alone and comes alone to church, power is husbanded, besides the very great advantage of a greater mental concentration on the subject of the discourse, and those affections and feelings of responsibility which ought to reign dominant when a man feels that he may be delivering his high message for the last time, or that for the last time it will come to some hearer, and, if not improved, will allow his unchangeable doom to be sealed—forever!

Any conscientious hearer of the word will find by experiment, that if the time up to the morning service is spent in quietude of body and mind, he will sing the first hymn with more alacrity and will enjoy it more deeply than if he had sung several hymns before, or had been engaged in a way to require bodily or mental effort.

When one, two or three hours only intervene between sermons, nothing should be eaten but some cold bread and butter, with a cup or two of any kind of hot drink; the former not to feed, but to sustain; the latter to impart the stimulus of warmth to the whole system. If a sermon is to be preached soon after a hearty meal, both speaker and hearer will be sleepy, while the mental effort necessary to deliver the discourse, withdraws so much of nervous power from the stomach that the food cannot be properly digested; and when repeated as a habit, chronic dyspepsia is engendered to burden the body and depress the mind for the remainder of life. These are not mere theories, but are from the experience of one who nearly a quarter of a century ago was able for two or three times a day, for months in succession, to speak extemporaneously, without apparent effort.

